# Effects of a rehabilitation program for individuals with chronic spinal cord injury in Shanghai, China

**DOI:** 10.1186/s12913-020-05181-x

**Published:** 2020-04-15

**Authors:** Fengshui Chang, Qi Zhang, Haixia Xie, Yuhui Yang, Mei Sun, Airong Wu, Jinghua Wu, Gang Chen, Feng Shen, Chengyue Li, Jun Lu

**Affiliations:** 1 China Research Center on Disability, School of Public Health, Fudan University, Shanghai, China; 2 School of Community and Environmental Health, Old Dominion University, Norfolk, VA USA; 3 Shanghai Yangzhi Rehabilitation Hospital (Shanghai Sunshine Rehabilitation Center), Shanghai, China; 4 Shanghai Disabled Persons’ Federation, Shanghai, China

**Keywords:** Chronic spinal cord injury, Rehabilitation, Rehabilitation effectiveness, Rehabilitation centers, China

## Abstract

**Background:**

Specialized Institution-Based Rehabilitation (SIBR) is the cornerstone of care and treatment for individuals with spinal cord injury, but most people with chronic spinal cord injury (CSCI) living in China have no SIBR experience after acute care hospital discharge. In 2009, an SIBR facility was set up in Shanghai (China) to fill this important gap in care. The purpose of the study was to evaluate the effectiveness of an integrated rehabilitation training program among individuals with CSCI living in Shanghai.

**Methods:**

A within-subject pre-posttest design was used to evaluate the SIBR. The sample included 455 individuals ≥1 year post-SCI, who were older than 18 years of age and were enrolled in a rehabilitation center in Shanghai, China, between 2013 and 2019. The data included individuals’ sociodemographic and injury characteristics, and twenty-three indicators were used as outcome measurements to evaluate basic life skills and their applications in family and social life. Multivariate linear regression was conducted to determine which factors might have influenced the effectiveness of the SIBR.

**Results:**

All basic life skills and their applications in family and social life were improved, but with variations across socio-demographics. Female individuals with CSCI had better outcomes in basic life skills than did males. In terms of basic life skills and their applications in family and social life, individuals with a low level (thoracic or lumbosacral) of injury achieved more significant functional gains than those with a higher level (cervical). The baseline score was also a relevant factor in functional outcome.

**Conclusions:**

Even for individuals with a long SCI history, SIBR training can improve basic life skills and the applications of those skills in family and social life settings.

## Background

Spinal cord injury (SCI) is defined as an injury in which the structures and function of the spinal cord are damaged due to traumatic or non-traumatic causes [1]. It is estimated that approximately 1.3 million individuals with SCI lived in China in 2012, and the reported incidence rates were 13–60 per million [2]. Although SCI is not curable [3], an individual’s Quality of Life (QoL) can be improved with timely, reasonable, and comprehensive rehabilitation services [4]. It is clear that rehabilitation training should start right after the injury to achieve better health outcomes for individuals with SCI [4–6]. However, emerging research suggests that late-start rehabilitation can still be effective [7, 8]. In developed countries, most individuals with SCI are admitted to inpatient rehabilitation centers directly from acute care [9] and typically receive at least 2–3 months of rehabilitation treatment [10]. Thereafter, they can continue to receive on-going family-supported rehabilitation, community rehabilitation, or specialized institution-based rehabilitation (SIBR) [11] with the aim of prevention of complications, family and community integration, etc. However, as a developing country, China has limited resources to provide rehabilitation services to individuals with SCI [12, 13]. They can receive some hospital-based rehabilitation services after the onset of injury that focus on body function improvement, complication prevention education, muscle strength exercise, and range of motion (ROM) training, then they return home with limited information and skills necessary for maintaining optimal health and well-being.

Before 2009, however, most people with SCI did not have access to any services after acute care hospital discharge. In 2009, the China Association of Persons with Physical Disability, a government-supported national organization, started community co-op centers called “halfway houses” for individuals with SCI in four provinces: Shanghai, Zhejiang, Henan, and Guangxi [14]. Halfway houses are the platform for community-based rehabilitation (CBR) for individuals with SCI. To better serve the individuals in Shanghai, an inpatient training center was set up that same year in the Shanghai Yangzhi Rehabilitation Hospital (Shanghai Sunshine Rehabilitation Center, SSRC), the only city-level specialized rehabilitation center in that province. The facility is responsible for providing SIBR training for individuals with SCI who need to improve their QoL and reintegrate into their communities, with all costs being paid by the Shanghai Disabled Persons’ Federation, a semi-government organization, which helps to ensure affordability, availability, and ease of administration. Chang et al. [14] reported that the mean time from injury to halfway house enrollment was 12.8 years in Shanghai between 2009 and 2015, which suggested that a majority of the enrollees had been managing their injuries for a long time without benefit of timely rehabilitation services. Considering the severity of the injury in those with SCI, SIBR should be the cornerstone of rehabilitation [15].

There has been extensive research into SCI rehabilitation in developed countries [16, 17]. However, existing research on SCI rehabilitation in China focuses on acute or sub-acute hospital-based interventions [18–20], and little research has examined the effectiveness of CBRs in China [21, 22]; to the best of our knowledge, the effects of an SIBR service for individuals with chronic spinal cord injury (CSCI) have not yet been studied in China. This study aimed to evaluate the effectiveness of the SIBR program for individuals with CSCI, meaning those who had lived with SCI for at least 1 year, at the training center in the SSRC and answered two questions: (1) What is the effect of rehabilitation on basic life skills and their applications in family and social life? (2) What are the factors that may change rehabilitation effectiveness after controlling for an individual’s functional level at admission to rehabilitation? We hypothesized that individuals with CSCI would likely experience therapeutic gains by participating in the program in the SSRC. We also investigated whether demographic and injury factors predicted rehabilitation effectiveness, for example, whether individuals with low levels of injury might acquire greater functional improvement compared with those with high levels of injury, and whether a shorter time since injury onset to admission in an SIBR program could bring better outcomes, etc. The study provides initial evidence in support of the establishment of further facilities and services in Shanghai and other developed regions in China to improve the welfare of individuals with SCI.

## Methods

### Participants

For the present study, a within-subject pre-posttest design was used. Inclusion criteria for participants included: (a) adult (between 18 and 70 years old), and (b) had lived with SCI for at least 1 year. Adults with severe medical co-morbidities, cognitive impairment, or mental disability were ineligible. Due to safety considerations and limited resources, the training program in the SSRC had no ability to provide services for individuals with tetraplegia whose injury level was higher than C5 (the fifth segment of the cervical spinal cord). A total of 455 individuals with CSCI who participated in rehabilitation training sessions in the SSRC between 2013 and 2019 were recruited into the study. These individuals were enrolled in the study from communities throughout 16 districts in Shanghai. Informed consent was obtained before arriving at the SSRC and was recorded on admission.

### Description of the intervention

To ensure the quality of rehabilitation training, the SSRC rehabilitation team designed an integrated rehabilitation training program based on the “biopsychosocial model” of disability of the International Classification of Functioning, Disability, and Health (ICF) [23] that focuses on the improvement of physical, psychological, and social functions of individuals with SCI. The contents of the program mainly included rehabilitation education and function reconstruction training in physical, psychological, and social functions [24–26].

Rehabilitation education covers an introduction to the spine and spinal cord, SCI and its rehabilitation, prevention of complications, bladder and bowel management, adaption and use of assistive devices, and strategies for creating barrier-free housing and environments. Body function reconstruction training includes physical therapy (PT) and occupational therapy (OT), which deals with muscle strength exercise, ROM, activities of daily living (ADLs), and wheelchair skills training, etc. Rehabilitation nursing training includes bladder and bowel management, personal hygiene, and the management of skin and body position. Psychological adjustment training consists of self-recognition, handling of emotions, interpersonal relationships, and social life adaptation after injury.

During the 45-day inpatient training period, individuals with SCI stayed in the SSRC and received training at different locations in or outside of the facility. Outside locations included supermarkets, movie theaters, restaurants, and subway stations, which were used to improve the social adaptability of patients. Due to the limited space in the SSRC training center, no more than 20 individuals were enrolled in each training session. A total of six training sessions were held each year. Every individual with SCI received a baseline evaluation in the first days after admission, after which the training was individualized according to the neurological level of injury, personal needs, and baseline function evaluation results. Personal needs were assessed by interview and group discussion by the training team. Each individual received a personalized schedule, which normally included 2 h of training (one session) in the morning and 3 h of training (two sessions) in the afternoon. Each person received a total of 90 training and education sessions (physical: 52 sessions / 84 h; social: 26 sessions / 44 h; psychological: 6 sessions / 12 h; summary and evaluation: 6 sessions / 10 h), with total sessions lasting for 150 h. Training and education methods included group discussion, demonstrations, role-playing, and practice.

The training team consisted of rehabilitation doctors and nurses, occupational therapists, physical therapists, psychological therapists, and social workers who had received a relevant bachelor’s degree or above and who had more than 5 years of SCI rehabilitation-related work experience. They received a two-week training before administering the intervention. In each term, two peer mentors joined the team as teaching assistants. These teaching assistants were required to have completed at least one training session successfully and to be able to facilitate communications between the SSRC rehabilitation team and individuals with SCI. Moreover, these teaching assistants were role models for newly enrolled trainees and helped the trainees learn rehabilitation skills in a timely fashion via demonstration.

### Data and measurement

This study used the same data and outcome measures as our previous study that has been published [27]. The evaluation tables and criteria are attached in the Additional file 1.

### Statistical analysis

Descriptive statistics and frequencies were calculated for socio-demographic and impairment characteristics. The Wilcoxon test of paired samples was used to evaluate the significance of the differences in median admission and discharge scores on basic life skills and their applications in family and social life [28]. Six separate multivariate linear regression models were run for our outcome measures. The dependent variables were the motor score, cognition and emotion score, total score on basic life skills, applications of basic life skills in family and social life score, and the total score of the basic life skills applications in family and social life. We included seven independent variables in the regressions. To control for the confounding effect of baseline-to-discharge scores [29], the baseline score of the dependent variable was added in the regression. Except for age on admission, time from injury to beginning rehabilitation training, and the baseline score of the dependent variable, all other independent variables were dichotomized: sex (0 = male; 1 = female), educational background (0 = junior high school or below; 1 = senior high school/secondary vocational school/junior or regular college), marital status (0 = single/divorced/widowed; 1 = married/common-law), neurological level of injury (0 = cervical cord; 1 = thoracic or lumbosacral cord), and etiology (0 = non-traumatic; 1 = traumatic). All statistical tests were two-sided. A *p*-value less than 0.05 was considered statistically significant, and no adjustments for multiple comparisons were made. The Statistical Package for Social Sciences for Windows (SPSS for Windows 13.0, SPSS Inc., Chicago, IL, USA) was used to analyze the data.

## Results

### Sociodemographic and impairment characteristics

The demographic and injury characteristics of the participants are presented in Table 1. Among the 455 individuals with CSCI, the male: female ratio was 1.76:1. The mean age (± SD) on admission was 46.2 ± 13.1 years. The mean age at injury (± SD) was 35.3 ± 15.2 years. The mean time (± SD) from injury to beginning rehabilitation training in the SSRC was 10.9 ± 12.2 years.

**Table 1:** Personal and injury characteristics of 455 individuals with chronic SCI

Variables	Numbers	Percentage (%)
Sex		
Male	290	63.7
Female	165	36.3
Education^a^		
Junior high school or below	287	63.1
Senior high school/secondary vocational school	92	20.2
Junior or regular college	76	16.7
Marital status^a^		
Unmarried	121	26.6
Married	283	62.2
Divorced or widowed	51	11.2
Levels of injury		
Cervical cord	135	29.7
Thoracic cord	241	53.0
Lumbosacral cord	79	17.4
Year of injury		
1953–2008	208	45.7
2009–2018	247	54.3
Etiology		
Non-traumatic	94	20.7
Traumatic	361	79.3

^a^: on admission; *SD* standard deviations

### Functional improvement of basic life skills and their applications in family and social life

**Fig. 1:**
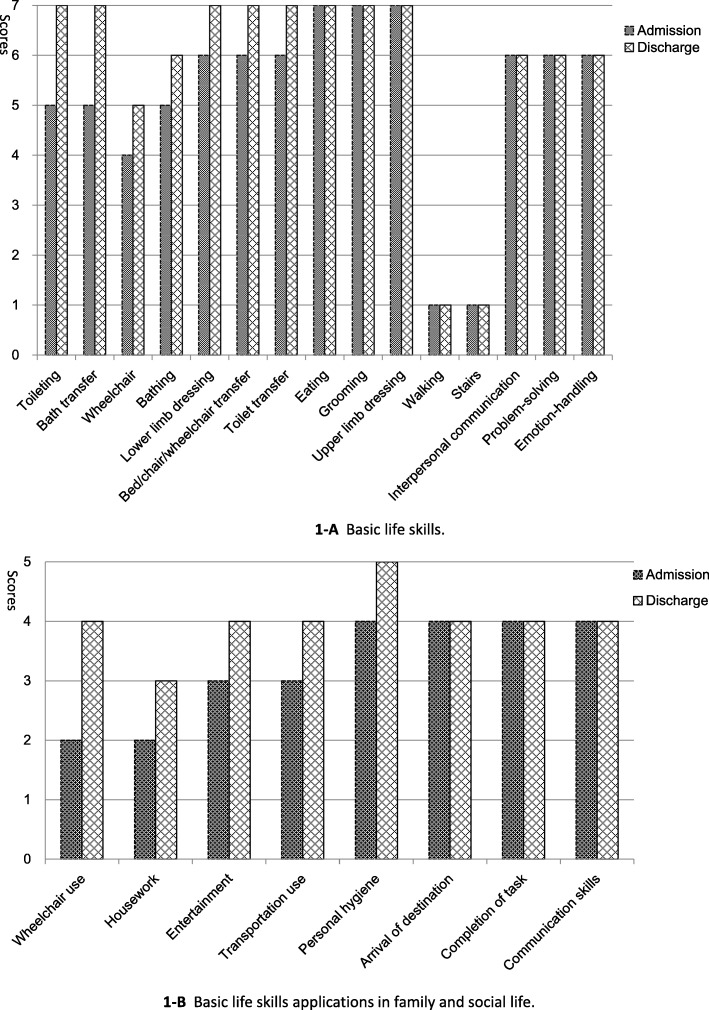
Bar charts showing admission and discharge scores in basic life skills and their applications in family and social life by individual variable (**a**: Basic life skills; **b**: Basic life skills applications in family and social life)

The median total basic life skills score increased by 14.1% between pre-integrated rehabilitation training to post-training (*p* <  0.01, Table 2), and the median total score of motor function increased to 70 at discharge from 61 on admission, with statistical significance. However, the median total cognition and emotion score was fixed at 18 from admission to discharge, which may be related to the high baseline score. Bar charts (Fig. 1) are used to show the median scores on admission and discharge for each item of the evaluation of basic life skills and their applications in family and social life. Among all skills, the median score increase was the greatest in toileting (40%) and bath transfer (40%, Table 2 and Fig. 1-a). For wheelchair (locomotion), bathing, upper limb dressing, toilet transfer and bed/chair/wheelchair transfer, there was an increase of 1 point (16.7–25.0%). However, the median scores did not increase for eating, grooming, or lower limb dressing because of a ceiling effect (Table 2 and Fig. 1-a). For the three cognition and emotion items, the median scores also did not increase. Owing to the fact that the majority of individuals with CSCI in this study were wheelchair dependent, the median scores of walking and use of stairs were fixed at 1. In addition to walking and using stairs, the score of wheelchair use (locomotion) at discharge ranked last (Table 2 and Fig. 1-a) among all basic life skills.

**Table 2:** Admission and discharge scores of basic life skills and their applications in family and social life, by individual variables

Variables	Median scores (range)			*p* value ^##^
	Admission	Discharge	Change (%)^#^	
A. Eating	7 (1–7)	7 (1–7)	0.0	< 0.01
B. Grooming	7 (1–7)	7 (1–7)	0.0	< 0.01
C. Bathing	5 (1–7)	6 (1–7)	20.0	< 0.01
D. Upper limb dressing	6 (1–7)	7 (1–7)	16.7	< 0.01
E. Lower limb dressing	7 (1–7)	7 (1–7)	0.0	< 0.01
F. Toileting	5 (1–7)	7 (1–7)	40.0	< 0.01
G. Bed/chair/wheelchair transfer	6 (1–7)	7 (1–7)	16.7	< 0.01
H. Toilet transfer	6 (1–7)	7 (1–7)	16.7	< 0.01
I. Bath transfer	5 (1–7)	7 (1–7)	40.0	< 0.01
J. Walking (locomotion)	1 (1–7)	1 (1–7)	0.0	< 0.01
K. Stairs (locomotion)	1 (1–7)	1 (1–7)	0.0	< 0.01
L. Wheelchair (locomotion)	4 (1–7)	5 (1–7)	25.0	< 0.01
Total motor score (range: 12–84)	61 (12–82)	70 (13–83)	14.8	< 0.01
M. Interpersonal communication	6 (1–7)	6 (1–7)	0.0	< 0.01
N. Problem-solving	6 (1–7)	6 (1–7)	0.0	< 0.01
O. Emotion-handling	6 (1–7)	6 (1–7)	0.0	< 0.01
Total cognition and emotion score (range: 3–21)	18 (3–21)	18 (3–21)	0.0	< 0.01
Total basic life skills score (range: 15–105)	78 (24–100)	89 (31–103)	14.1	< 0.01
P. Personal hygiene	4 (1–5)	5 (1–5)	25.0	< 0.01
Q. Housework	2 (0–5)	3 (0–5)	50.0	< 0.01
R. Entertainment	3 (0–5)	4 (0–5)	33.3	< 0.01
Total score of basic life skills applications in family life (range: 0–15)	8 (1–15)	11 (1–15)	37.5	< 0.01
S. Wheelchair use	2 (0–5)	4 (0–5)	100.0	< 0.01
T. Transportation use	3 (0–5)	4 (0–5)	33.3	< 0.01
U. Arrival at destination	4 (0–5)	4 (1–5)	0.0	< 0.01
V. Completion of task	4 (0–5)	4 (2–5)	0.0	< 0.01
W. Communication skills	4 (1–5)	4 (2–5)	0.0	< 0.01
Total score of basic life skills applications in social life (range: 0–25)	17 (3–25)	20 (9–25)	17.6	< 0.01
Total application score (range: 0–40)	25 (5–40)	31 (13–40)	24.0	< 0.01

#: Relative improvement = (Discharge – Admission)/Admission * 100

##: Wilcoxon test of paired samples was performed

The scores of basic life skills applications in family and social life improved significantly (*p* <  0.01, Table 2) from pre- to post-test, and the percentage increase in the median total scores of basic life skills applications in family and social life was 37.5 and 17.6%, respectively. The increase among the eight indicators of family and social life score was the greatest in wheelchair use (100.0%, Table 2 and Fig. 1-b) and housework (50.0%); however, the discharge score was the lowest in housework, with a 1-point increase (Table 2 and Fig. 1-b).

By examining the range of the scores (Table 2), we noticed that after rehabilitation training, the lowest scores of some items of basic life skills applications in social life increased by 1 to 2 points, such as arrival at destination and completion of tasks. However, the scores of many more items were still low, such as all the motor skills, housework, and wheelchair use, which indicated that rehabilitation training may not have improved basic life skills and their applications in family and social life in some individuals with CSCI.

### Related factor analyses of functional improvement

The regression results showed that sex and the level of injury were significant predictors of improved motor scores (Table 3). Female individuals with CSCI showed greater improvement than male peers in basic life skills (motor: β = 0.12, *p* = 0.01; cognition and emotion: β = 0.08, *p* = 0.046). Compared with individuals with a high neurological level of injury, the motor score of individuals with low injury level (thoracic and lumbosacral cord) increased more significantly (β = 0.32, *p* <  0.01). None of the factors except for sex and baseline score were related to cognitive and emotional score improvement (*p* > 0.05). For basic life skills as a whole, the pattern was similar to motor function, where sex and level of injury were the two related factors. The results showed that the age, time span since injury, educational background, etiology and marital status were not significant predictors of functional improvement in basic life skills (*p* > 0.05). The proportions of the variance (R^2^) explained by the models were 34% (motor score), 39% (cognition and emotion score), and 35% (total basic life skills score), separately.

**Table 3:** Linear regression analyses of determinants of functional improvement in basic life skills of individuals with chronic SCI

Independent variables	Motor score			Cognition and emotion score			Total score		
	β	t-value	*p* value	β	t-value	*p* value	β	t-value	*p* value
Age^a^	0.06	1.16	0.25	−0.10	−1.91	0.06	0.02	0.31	0.75
Time from injury to beginning rehabilitation training	−0.06	− 1.20	0.23	0.02	0.50	0.62	−0.04	− 0.81	0.42
Sex									
Male	Reference			Reference			Reference		
Female	0.12	2.76	0.01	0.08	2.01	0.046	0.11	2.49	0.01
Education^a^									
Junior high school or below	Reference			Reference			Reference		
Senior high school or higher	−0.004	−0.09	0.93	−0.03	−0.73	0.47	−0.07	−1.44	0.15
Marital status^a^									
Unmarried	Reference			Reference			Reference		
Married	0.06	1.11	0.27	0.01	0.12	0.90	0.07	1.34	0.18
Etiology									
Non-traumatic	Reference			Reference			Reference		
Traumatic	−0.01	−0.29	0.78	0.05	1.21	0.23	−0.01	−0.18	0.86
Levels of injury									
Cervical cord	Reference			Reference			Reference		
Thoracic and Lumbosacral cord	0.23	4.97	< 0.01	−0.06	−1.40	0.16	0.22	4.33	< 0.01
Baseline score	−0.56	− 12.84	< 0.01	− 0.64	− 15.61	< 0.01	− 0.58	− 12.59	< 0.01
	Adjusted R^2^ = 0.32 (F = 23.44; *p* < 0.01)			Adjusted R^2^ = 0.37 (F = 36.32; *p* < 0.01)			Adjusted R^2^ = 0.32 (F = 22.23; *p* < 0.01)		

^a^: on admission

With regards to basic life skills applications in family and social life, thoracic and lumbosacral cord injury was the only factor related to the effect of training (*p* <  0.01, Table 4); i.e., the individuals with a lower injury level achieved more improvement in the applications of basic life skills in family and social life than did individuals with a higher level of SCI. However, six other factors also affected the functional outcome of basic life skills applications in family and social life, though without significance (Table 4). Moreover, the baseline score of basic life skills and their applications in family and social life was also a related factor (*p* <  0.01). The proportions of the variance explained by the models were 40% (family life), 39% (social life), and 39% (total application score), separately.

**Table 4:** Linear regression analyses of determinants of functional improvement of basic life skills applications in family and social life

Independent variables	Family life			Social life			Total score		
	β	t-value	*p* value	β	t-value	*p* value	β	t-value	*p* value
Age^a^	0.01	0.21	0.83	−0.03	−0.70	0.48	−0.002	− 0.04	0.97
Time from injury to beginning rehabilitation training	−0.04	−0.94	0.35	−0.03	− 0.60	0.55	− 0.06	− 1.33	0.18
Sex									
Male	Reference			Reference			Reference		
Female	0.01	0.19	0.85	−0.01	−0.16	0.88	0.01	0.30	0.77
Education^a^									
Junior high school or below	Reference			Reference			Reference		
Senior high school or higher	0.001	0.02	0.98	0.05	1.29	0.20	0.04	1.07	0.29
Marital status^a^									
Unmarried	Reference			Reference			Reference		
Married	0.06	1.33	0.19	0.06	1.31	0.19	0.07	1.39	0.17
Etiology									
Non-traumatic	Reference			Reference			Reference		
Traumatic	−0.03	−0.75	0.46	−0.05	−1.32	0.19	−0.05	−1.37	0.17
Levels of injury									
Cervical cord	Reference			Reference			Reference		
Thoracic and Lumbosacral cord	0.21	4.82	< 0.01	0.11	2.80	0.01	0.18	4.17	< 0.01
Baseline score	−0.59	−13.70	< 0.01	− 0.65	−16.67	< 0.01	− 0.60	−14.43	< 0.01
	Adjusted R^2^ = 0.38 (F = 27.01; *p* < 0.01)			Adjusted R^2^ = 0.37 (F = 39.27; *p* < 0.01)			Adjusted R^2^ = 0.37 (F = 31.20; *p* < 0.01)		

^a^: on admission

## Discussion

The goal of this study is to assess the effectiveness of a novel SIBR training program in China designed to improve outcomes in individuals with CSCI. Our results suggest that SIBR training is an effective intervention to improve abilities in basic life skills and their applications in family and social life, even for individuals with a long SCI history [7]. Wheelchair use, housework, toileting, and bath transfer were the four items that showed relatively the most improvement. However, the gains in skills were not consistent across all domains, and some family and social skill items still needed further attention. The results were consistent with a previous study in Australia [16], which described significant motor FIM score improvement in inpatient rehabilitation for adults of SCI. Of course, the fact that scores did not change for eating, grooming, walking, and using stairs is to be expected [16, 30]. More research is needed to understand how to improve these scores further within the SIBR and CBR settings.

Female individuals with CSCI acquired more improvement in basic life skills than their male counterparts. The possible reason is that women may have more natural neurologic recovery than men [31]. However, Teeter et al. reported that being male was the predictive variable for the motor FIM score at discharge from initial rehabilitation and at 1 year post-injury for patients with paraplegia [32].

Individuals with thoracic and lumbosacral cord injuries had a better outcome in motor function and basic life skills applications in family and social life than those with cervical cord injuries. Similar results were seen in a study of rehabilitation hospitals in the USA, where the level was the predictor of motor FIM at discharge and 1-year post-injury [33].

Studies from Canada [34] and the USA [33] revealed that injury age and time from injury to rehabilitation admission were negative predictors to discharge, 1-year and 2-year post-injury FIM scores. However, this study suggested that age on admission was a factor related to motor score, though with no statistical significance, and the same was true for the time from injury to rehabilitation admission.

Both this study and one conducted in India [35] reported there was statistically no significant difference in the functional outcome between individuals with traumatic and non-traumatic SCI after inpatient rehabilitation. However, a previous study has shown that patients with traumatic SCI achieved greater overall functional improvement than non-traumatic SCI in the USA within 60 days of injury [36]. The current study demonstrated that educational background on admission was not related to functional outcome of SIBR for individuals with CSCI. However, a previous study has shown that higher pre-injury education levels predicted higher FIM scores at 1 year post-injury [32].

This study has implications for policy makers. First, for individuals with SCI, it is never too late to start rehabilitation. People with SCI should be given on-going rehabilitation service to help them enjoy a long and full life [4, 37]. Our research provided further evidence of the value of late-start rehabilitation [7, 8], since individuals with SCI in our study had been injured an average of 10.9 years earlier but still improved in certain functions after rehabilitation training. Since Shanghai’s GDP per capita reached USD$20,000 in 2018, making it equivalent to the threshold level of the developed economies, the city government could conceivably reallocate more resources to provide rehabilitation training for more individuals with SCI. Hospitals may then refer newly injured individuals with SCI to the SSRC to receive timely rehabilitation training after discharge, and individuals with SCI in communities who have not participated in rehabilitation training can also be recommended to the SSRC in a timely manner. A model facilitating referral and coordination between hospitals, specialized rehabilitation institutions, and communities is essential for fulfilling this goal [38, 39]. In short, this study is based on a representative sample of individuals with CSCI participating in rehabilitation training at the SSRC. Findings from this study will help the SSRC as well as the Shanghai government and other developed regions to allocate more resources in the SIBR area.

The second implication of this study is that the long-term goal of rehabilitation is always to promote social inclusiveness and help individuals with SCI reintegrate into their society [40]. Although the direction of change for individuals with SCIs in China has been positive, we still have a long way to go to meet the needs of current and future individuals with this condition [41–43].

Finally, after the participants in this study received integrated rehabilitation training in the SSRC, they were able to share their experiences and basic life skills with other individuals with SCI in their local communities, which would tend to strengthen the community-wide effects of rehabilitation training [44, 45]. Newly injured individuals with SCI might apply for admission to rehabilitation training in the SSRC under the influence of our trainees.

This study has a few limitations. First, the sample size was limited due to the relatively low incidence of SCI and the limited service capacity of the SSRC training center. Therefore, the factors that did not demonstrate statistical significance in this study should not be excluded from future research. Second, the explained variance (R-square) was relatively low for regression analyses. This could be partly traced to the fact that because of limited resources for data collection, we could not obtain information on training processes, caregivers, and other social supports or follow-up outcomes. More independent variables and larger sample sizes could result in a higher R-square. Third, the severity of injury based on ASIA (American Spinal Injury Association) classification was not collected. Fourth, this study was a pre-post study, so its external validity is limited. A more rigorous study design, such as a randomized controlled trial (RCT), should be implemented to establish causal relationships.

## Conclusions

In summary, this study evaluates the rehabilitation outcomes of 455 individuals with CSCI who participated in integrated rehabilitation training in the SSRC. Our results suggest that even for individuals with a long SCI history, rehabilitation training can still improve their basic life skills and the applications of those skills in family and social life. Therefore, our study provides preliminary evidence to support the value of SIBR for individuals with SCI in Shanghai. Since many other regions in China have reached a level of economic development similar to Shanghai, the model in Shanghai could be recommended for those regions.

## Supplementary information

**Additional file 1.** Evaluation Tables and Criteria

## Data Availability

The datasets analysed during the current study are not publicly available due to our data policy, but are available from the corresponding author on reasonable request.

## Abbreviations

SCI: Spinal cord injury
CSCI: Chronic spinal cord injury
CBR: Community-based rehabilitation
SSRC: Shanghai Sunshine Rehabilitation Center
SIBR: Specialized Institution-Based Rehabilitation
ICF: International Classification of Functioning, Disability and Health
PT: Physical therapy
OT: Occupational therapy
ROM: Range of motion
ADL: Activities of daily living
FIM: Functional independence measurement
SD: Standard deviations
RCT: Randomized controlled trial
QoL: Quality of life
ASIA: American Spinal Injury Association
