# Supplementary material for: Effects of a rehabilitation program for individuals with chronic spinal cord injury in Shanghai, China
Source: BMC Health Serv Res. 2020 Apr 15;20:298. doi: 10.1186/s12913-020-05181-x (PMC7158161; doi:10.1186/s12913-020-05181-x)
Supplement: Supplementary file 1 — Additional file 1. Evaluation Tables and Criteria [file 12913_2020_5181_MOESM1_ESM.pdf]

Evaluation Table I. Basic Life Skills

Name of case______________ Evaluator_______________

Items		Admission	Discharge
		Date:_______________	Date:_______________
		7 6 5 4 3 2 1	7 6 5 4 3 2 1
Self-care	Eating		
	Grooming		
	Bathing		
	Lower limb dressing		
	Upper limb dressing		
	Toileting		
Transfer	Bed/chair/wheelchair transfer		
	Toilet transfer		
	Bath transfer		
Locomotion	Walking		
	Stairs		
	Wheelchair		
Cognition and emotion	Interpersonal communication		
	Problem-solving		
	Emotion handling		
Total scores (range:15-105 scores):			

Evaluation Criteria for Basic Life Skills

Functional classification	Score	Descriptions
Complete independence	7	The activity can be completed normatively within reasonable time and without any modification, assistive device or any help.
Modified independence	6	The activity can be completed without any help from others, but assistive device is required (artificial limbs and braces, etc.), or it takes longer time than normal to complete it, or is not performed safely.
Partial dependence	5	Supervision or setup: required help is only limited to spare, remind or advice; there is no physical touch between a helper and a patient; or a helper only needs to help prepare necessities; or help with an orthosis.
	4	Minimal contact assistance: the help provided for a patient is only limited to light contact, and a patient can complete 75% or more task independently.
	3	Moderate contact assistance: the help provided for a patient goes beyond light contact, and a patient can complete 50%-74% task independently.
Complete dependence	2	Maximal assistance: a patient can complete 25%-49% task independently, that is, the subject needs over half help, otherwise, the task cannot be carried out.
	1	The effort made by a patient is lower than 25%, that is, a patient can only contribute to less than 1/4 in completing the task.

Evaluation Table II. Applications in Family and Social Life

Name of case____________ Evaluator ____________

Items	Admission	Discharge
	Date:	Date:
I. Application in family life		
1. Personal hygiene	0 1 2 3 4 5	0 1 2 3 4 5
2. Housework	0 1 2 3 4 5	0 1 2 3 4 5
3. Entertainment	0 1 2 3 4 5	0 1 2 3 4 5
II. Application in social life		
1. Wheelchair use	0 1 2 3 4 5	0 1 2 3 4 5
2. Transportation use	0 1 2 3 4 5	0 1 2 3 4 5
3. Arrival of destination	0 1 2 3 4 5	0 1 2 3 4 5
4. Completion of task	0 1 2 3 4 5	0 1 2 3 4 5
5. Communication skills	0 1 2 3 4 5	0 1 2 3 4 5
Total scores: (range: 0-40 scores)		

Evaluation Criteria for Applications of Basic Skills in Family and Social Life

Type	Indicators	Evaluation points	5	4	3	2	1	0
Application in family life	Personal hygiene	Wash the face, brush teeth, take a shower, trim nails, shave, conduct skin care, keep oral hygiene, dining hygiene, toilet hygiene, wear and match clothes etc.	Good personal hygiene, dressing properly	Good personal hygiene, and good personal image	So-so personal hygiene, and be able to realize basic self-care	So-so personal hygiene, and need the help of others	Poor personal hygiene, and need the help of others	Poor personal hygiene, and need the help of others
	Housework	Clean the room, sort out articles, wash and hang out clothes, boil and pour water, make preprandial preparations, cook foods, conduct postprandial processing and safely use water, fire, electricity and gas etc.	Be able to do many housework, and even do some of it better than normal people	Be able to do many housework, and do as well as normal people	Enjoy doing housework and be able to do some difficult one	Be willing to do housework and do it frequently, except for too difficult one	Be willing to do housework and do it occasionally	Never do it and be reluctant to do it
	Entertainment	Listen to the radio, watch films and TV, sing,	Know leisure and entertainment methods well, and	Know leisure and entertainment methods well, and	Enjoy participating in some leisure and recreation activities	Be willing to and frequently participate in	Occasionally participate in some leisure and	Never participate in leisure

		play, use musical instruments, weave, play cards, play chess, surf the Internet, exercise and keep fit, and travel etc.	be able to organize collective activities	be able to regulate physical and mental health		some leisure and entertainment activities	entertainment activities	and entertainment activities
Application in social life	Wheelchair use	Drive on various road surfaces, go up and down a ramp, stride over obstacles, go up and down stairs	Independently use a wheelchair	Know use skills of a wheelchair well, be able to expertly use a wheelchair to complete ground driving (over 30 min), go up and down a ramp and stride over obstacles	Know use skills of a wheelchair well, be able to expertly use a wheelchair to complete ground driving (30 min)	Be able to use a wheelchair to complete ground driving for 20 minutes	Be willing to use a wheelchair, but have a poor ability to use, and only be able to complete short-time ground driving (5 min)	Fail to independently use a wheelchair.
	Transportation use	Take a taxi, subway, light rail, train or airplane etc.	Dare to and be able to independently take a taxi, subway, light rail, train or airplane etc., and do not need the help of others	Be able to take a taxi, subway, light rail, train or airplane etc. without the company of others, and be able to ask for help in case of any difficulty properly	Be able to take a taxi subway, light rail, train or airplane etc. with the company of others, and only need a little help	Be able to take a taxi, subway, light rail, train or airplane etc. with the company of others, and need more help	Be willing but be afraid to take a taxi, subway, light rail, train or airplane etc.	Cannot take any transportation means, and never try to ask for help
	Arrival of destination	Work unit, hospital, supermarket, shopping mall, bank, post office, restaurant,	Located outside the neighborhood, and needed to be reached by relevant vehicle, and be able to arrive at such destinations	Located outside the neighborhood,, and needed to be reached by relevant vehicle, and be able to arrive at such destinations	Located outside the neighborhood,, and needed to be reached by relevant vehicle, and be able to arrive at such destinations	Located in the neighborhood, and be able to arrive at such destinations safely,	Located in the neighborhood, and be able to arrive at such destinations safely, independently and effectively with a little	Cannot arrive at such destinations, or never try to do

		barbershop, railway station, airport and neighborhood etc.	safely, independently and effectively without the help of others basically, and usually go to more places	safely, independently and effectively without the help of others basically, and usually go to fixed places	safely, independently and effectively with a little help of others, and go to fixed places usually	independently and effectively without the help of others basically, and go to fixed places usually	help of others only, and go to fixed places usually	that
	Completion of task	Matter handling ability in a work unit, hospital, supermarket, shopping mall, bank, post office, restaurant, barbershop, train station, airport and community	Be able to independently complete tasks outside a community, such as going out to work, seeing a doctor , dining in a restaurant, going shopping and handling businesses	Be able to complete tasks outside a community with a little help of others such as going out to work, seeing a doctor, dining in a restaurant, going shopping and handling business.	Be able to independently complete tasks within a community such as going shopping in a supermarket, taking exercise and participating in activities in an activity room.	Be able to complete tasks within a community with a little help of others such as going shopping in a supermarket, taking exercise and participating in activities in an activity room	Be willing to but need more help to complete tasks within a community such as going shopping in a supermarket, taking exercise and participate in activities in an activity room	Cannot complete tasks or never try to do them
	Communication skills	Help seeking, expression, social etiquette, polite words and task completing etc.	Have a good communication ability and a clear language expression ability and be able to smoothly complete tasks by a correct help-seeking method	Have a good communication ability and a clear language expression ability, and be willing to try various help-seeking methods to complete tasks	Have a good communication ability and a clear language expression ability, and be able to complete tasks basically by a help-seeking method	Have a general communication ability and a language expression ability, be willing to ask for help, and be able to complete tasks basically	Have a slightly poor communication ability, and an unclear language expression ability, be willing to ask for help, but adopt an incorrect method, and poorly complete tasks	Have a poor communication ability, and be reluctant to communicate and express himself (herself)
